# Structural Dynamics Behind Clinical Mutants of PncA-Asp12Ala, Pro54Leu, and His57Pro of *Mycobacterium tuberculosis* Associated With Pyrazinamide Resistance

**DOI:** 10.3389/fbioe.2019.00404

**Published:** 2019-12-10

**Authors:** Aamir Mehmood, Muhammad Tahir Khan, Aman Chandra Kaushik, Anwar Sheed Khan, Muhammad Irfan, Dong-Qing Wei

**Affiliations:** ^1^The State Key Laboratory of Microbial Metabolism, College of Life Sciences and Biotechnology, Shanghai Jiao Tong University, Shanghai, China; ^2^Department of Bioinformatics and Biosciences, Capital University of Science and Technology, Islamabad, Pakistan; ^3^Wuxi School of Medicine, Jiangnan University, Wuxi, China; ^4^Department of Microbiology, Kohat University of Science and Technology, Kohat, Pakistan; ^5^Department of Microbiology and Cell Science, Genetics Institute and Institute of Food and Agricultural Sciences, University of Florida, Gainesville, FL, United States

**Keywords:** drug resistance, simulation, mutations, PZA, MTB

## Abstract

Pyrazinamide (PZA) is one of the main FDA approved drugs to be used as the first line of defense against *Mycobacterium Tuberculosis* (MTB). It is activated into pyrazinoic acid (POA) via MTB's *pncA* gene-encoded pyrazinamidase (PZase). Mutations are most commonly responsible for PZA-resistance in nearly 70% of the resistant samples. In the present work, MTB positive samples were chosen for PZA drug susceptibility testing (DST) against critical concentration (100 ug/ml) of PZA. The resistant samples were subjected to *pncA* sequencing. As a result, 36 various mutations have been observed in the PZA resistant samples, uploaded to the NCBI (GeneBank accession no. MH461111). Here we report the mechanism of PZA resistance behind the three mutants (MTs), Asp12Ala, Pro54Leu, and His57Pro in comparison with the wild type (WT) through molecular dynamics simulation to unveil how these mutations affect the overall conformational stability. The post-simulation analyses revealed notable deviations as compared to the WT structure. Molecular docking studies of PZA with MTs and WT, pocket volume inspection and overall shape complementarity analysis confirmed the deleterious nature of these mutations and gave an insight into the mechanism behind PZA-resistance. These analyses provide vital information regarding MTB drug resistance and could be extremely useful in therapy management and overcoming its global burden.

## Introduction

Tuberculosis (TB) is an infectious disease that mainly attacks the lungs and then spreads to other parts of the body such as the brain and spine and is caused by a type of bacteria called *Mycobacterium tuberculosis* (MTB). It is asymptomatic in its latent stage, however, there does exist a risk of its development into the active state during the lifetime of an individual. It is claimed to be the 9th leading cause of death globally in the reports published by the World Health Organization (WHO) in 2018. Roughly 1.3 million TB expiries happened in 2017 apart from 374,000 deaths (10%) among HIV-positive individuals amongst 10.4 million total TB cases (90% adults). Around 1.7 billion individuals (23% of the world's population) are calculated to have a latent TB infection, representing a threat of active TB development during their lifespan. Asian countries like India, Indonesia, China, Philippines, and Pakistan account for 56 % of the global TB burden (Floyd et al., 2018). MTB exists in the alveolar macrophages of the infected individuals in its latent state. However, only 5–10 % of this bacteria matures from dormant into its active form (Ai et al., 2016; Houben and Dodd, 2016). Furthermore, the chances of active TB development could be increased in the case of co-infections like TB-HIV, immunosuppressants and aging (Fujita, 2011; Bruchfeld et al., 2015; Mekonnen et al., 2015). Pyrazinamide (PZA) is the only drug that kills MTB in a latent state which has successfully reduced the period of TB therapy from 9 to 6 months (Mitchison, 1985; Yang et al., 2015; Yadon et al., 2017). PZA is a prodrug that depends on MTB encoded pyrazinamidase (PZase) activity for its conversion into pyrazinoic acid (POA), an active form of PZA that targets the trans-translational (Zhang et al., 2003; Lu et al., 2011) process of ribosomal protein S1 (RpsA). There the POA disrupts the formation of RpsA-tmRNA complex in MTB (Sørensen et al., 1998; Simons et al., 2013; Tan et al., 2014; Yang et al., 2015). This disruption has potential effects on the persistent forms of MTB (Stehr et al., 2015; Njire et al., 2016). PZA resistance is associated with mutations in multiple target genes, including *pncA, rpsA*, and *panD*. However, mutations in the *pncA* are the major mechanism of resistance in 70 to 97% cases with an average of 85% resistance in MTB isolates (Alexander et al., 2013; Zhang et al., 2013; Akhmetova et al., 2015; Khan et al., 2018a,b). PZase consists of four α-helices and six parallel β-sheets. The metal ion Fe^2+^ is surrounded by one aspartate (Asp49) and three histidine residues, His51, His57, and His71. The amino acids Asp8, F13, L19, V21, D49, W68, H71, F94, Y95, Lys96, Y103, I133, A134, H137, and C138 have been observed in the catalytic site and its outskirts (Du et al., 2001; Petrella et al., 2011). Genetic mutations frequently affect the electrostatic nature of the target protein surface that in turn may exert influence on molecular dynamics (MD), causing loss of drug binding affinity (Ma et al., 2003; Haq et al., 2012; Li et al., 2017; Aggarwal et al., 2018). Identification and analyses of mutations in the drug resistance MTB strains might be helpful to unveil the underlying molecular mechanisms for better management of the resistant TB.

Three major regions (amino acids 3 to 17, 61 to 85, and 132 to 142) of the *pncA* are most commonly affected by mutations associated with changes in the PZase catalytic activity (Lemaitre et al., 2001; Sheen et al., 2009). However, Yoon et al. (2014) reported that mutations occurred at far from the active site may affect protein activity by altering the expression levels or protein folding. Protein structures may be drastically be affected by amino acid substitutions that directly alter the function, especially those mutations that occur in the active site or binding pockets (Bartlett et al., 2003; Worth et al., 2009; Ganesan and Ramalingam, 2019). Such variations may also have effects on a long-range position (Kosloff and Kolodny, 2008). Exploring the mechanism of changes that occur behind a mutation is required for better understanding, however, such molecular investigations are time-consuming and excessively high to be addressed by experimental procedure alone.

Molecular dynamic (MD) simulations have been applied widely in exploring the mechanisms of conformational amendments in a protein, especially in drug resistance situations arisen as a result of mutations. MD simulation studies of ligand-protein interactions are widely applied approaches for explaining the mechanisms of drug resistance caused by genetic variations in the target protein. However, it can be confirmed by experimental conditions only as every protein-drug complex does not explore the mechanism of resistance, neither every structure can be attained by single-crystal X-ray diffraction. In comparison with the experimental approaches, MD simulation has a particular advantage of exploring the causes of drug confrontation at the molecular level (Liu and Yao, 2009). Furthermore, the structural dynamics of protein complexes and other residues' level information can be accessed which have been considered difficult by experimental procedures (Hou et al., 2008; Xue et al., 2012a,b; Ding et al., 2013).

In our recent studies, we have performed PZA drug susceptibility testing followed by the sequencing of the *pncA* gene and identified some novel mutations (Asp12Ala, Pro54Leu and His57Pro) of which the sequences have been submitted to the GeneBank (Accession No. MH461111) which were observed to be associated with PZA-resistance (Xue et al., 2012a; Khan et al., 2019a). We aimed to analyze the structural dynamics of WT and PZase mutations that are involved in metal binding or present in its surroundings. The obtained outcomes may be useful to enhance our understanding of the MTB drug resistance on the molecular level.

## Materials and Methods

### Ethical Considerations

The Institutional Ethics Committee of Cust Islamabad and Provincial Tuberculosis Reference Laboratory (PTRL) at Khyber Pakhtunkhwa (KP) Pakistan permitted the current study. A well-versed consent from each TB patient was taken before the initialization of this study, however, the obtained outcomes were not meant to reflect an individual patient.

### Study Sample

The obtained samples from TB patients as a whole were handled at the BSL-III facility of PTRL, Hayatabad Medical Complex (HMC). This laboratory accepts TB cases from the entire KP province where the MGIT 960 system is used for testing drug susceptibility. The study samples were provided by the courtesy of guardians or concierges.

### Sample Processing, Isolation, and Mycobacterial Culture

In order to process these samples, the N-acetyl-L-cysteine–sodium hydroxide (NALC–NaOH) concentration technique (Kubica et al., 1963) was used. An equal amount of NaOH/N-acetyl-L-cysteine (NALC) contained in a Falcon tube containing both these concentrations were vortexed subsequently. This whole setup was incubated for a duration of 15 min to be decontaminated and digested. Afterward, a phosphate buffer solution of 50 ml was poured into each tube and was centrifuged for 15 min at the rate of 3,000 rpm. A separate tube that contained 5% of phenol was used to transfer the supernatant, while the phosphate buffer was mixed with the pellet. The Lowenstein–Jensen medium (LJ) in MGIT tubes containing 7H9 media was used for culturing purposes.

### Drug Susceptibility Testing (DST)

The automated BACTEC MGIT 960 system (BD Diagnostic Systems, NJ, USA) resistance result with mutations have already been investigated (Siddiqi and Rüsch-Gerdes, 2006; Khan et al., 2019a) and were used to test the PZA drug susceptibility. The Mycobacterium tuberculosis H37Rv and Mycobacterium Bovis were used as vulnerable and resilient controls, respectively. All those samples were labeled as PZA resistant that showed a growth of 100 μg/ml of the PZA critical concentration. This process was repeated several times in order to confirm the PZA resistance. These resilient samples were again subjected to DST in addition to the ionized (INH), rifampin (RIF), ethanol (EMB), amikacin (AMK), streptomycin (SM), capreomycin (CAP), ofloxacin (OFX), and Kanamycin (KM) via the BACTEC MGIT 960 system. The optimum concentration of PZA was kept regarding the guidelines of WHO (Horne et al., 2013). The development of MTB against the drug concentration was investigated manually as well for validation purposes.

### DNA Extraction and PCR Amplification of PZA-Resistant Samples

All the PZA-resistant samples were subjected to genomic DNA extraction via sonication (Buck et al., 1992; Kirschner et al., 1993; Khan et al., 2019b). The Mycobacterium Growth Indicator Tube (MGIT) that contained a fresh culture from which 1 μL was taken and transferred to a microcentrifuge tube. An Echotherm™ IC22 Digital was used for boiling purposes at 86°C for a duration of 30 min. Next, to this step, sonication was carried out for 15 min via ELMASONIC S30 sonicator. After sonication, all the TB samples were subjected to centrifugation for 5 min at the rate of 10,000 rpm. As a result of centrifugation, the obtained supernatant contained DNA that was kept at −20°C. The previously testified primers (*pncA*-F = 5GCGTCATGGACCCTATATC-3 and *pncA*-R = 5 AACAGTTCATCCCGGTTC-3=) (Liu et al., 2018) were managed and used for the amplification of those fragments that contained the *pncA*.

For PCR, each 50-μl reaction was prepared by adding each forward and reverse primer of 1 μl, an individual DNT of 0.1 μl and MgCl2 of 3 μl. An amount of 0.8 μl of Taq (New England Biolabs, UK), molecular grade water of 34.8 μl along with a genomic DNA of 4 μl was also added on to the reaction. For a successful run of this PCR reaction, a temperature of 94°C for 5 min was kept for the sake of denaturation. Next, to this, 30 cycles were run for a time of 30 s with a temperature scale of 94°C, then again 30 s at 56°C, and lastly at 72°C for a duration of 1 min. As mentioned earlier, the extension step was conducted at 72°C for 5 min. These amplified PCR samples were submitted to 6 Applied Biosystems 3730xl (Macrogen Korea) for sequencing purposes.

### Sequenced *pncA*

The Mutation Surveyor V5.0.1 software (Dong and Yu, 2011) was applied to handle the sequenced data which was examined and equated with the parent *PncA* gene (Rv2043c) of NCBI (NC_000962).

### Data Mining and Preparation

The 3D crystallographic structure for MTB PZase was obtained from the Brookhaven Raster Display Protein Data Bank (PDB) by typing 3PL1 (Berman et al., 2000; Junaid et al., 2018). All the water molecules from the MTB protein structure were removed via PyMOL (Bhattacharya et al., 2018). The structure was saved as a wild while 3 more same copies were produced having mutations (Asp12Ala, Pro54Leu, and His57Pro) incorporated at the respective positions. The mutation builder tool implemented in the Molecular Operating Environment (MOE) software suite was used for mutating the structures.

**Figure 1:**
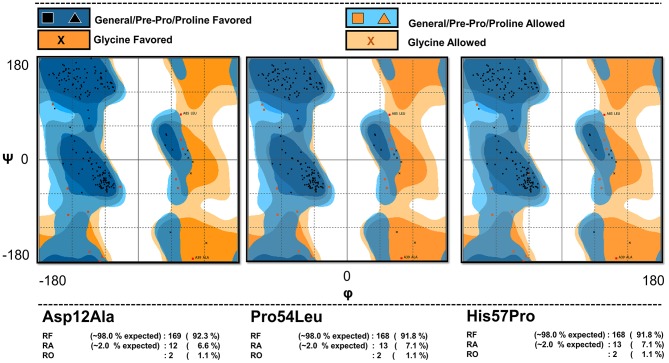
Model validation. The Ramachandran plot analysis of the mutant models showing the percentage of their residues in various regions confirming the validity of the mutated models.

The mutants' conformations were validated via Ramachandran plot analysis (Figure 1) hosted by the RAMPAGE server (http://mordred.bioc.cam.ac.uk/~rapper/rampage.php) (Lovell et al., 2003; Vedamurthy et al., 2019). The PubChem (Kim et al., 2015), an online database for chemical compounds was accessed to obtain the 3D chemical structure of PZA drug (PubChem CID: 1046) (Kim et al., 2015). PubChem is powered by the National Institutes of Health (NIH) and it serves as a bank for storing and sharing chemical structures that can be accessed at https://pubchem.ncbi.nlm.nih.gov/. The Chimera software package (Banu et al., 2018) was used for energy minimization of the protein-drug complexes before simulation to avoid any unnecessary atomic clashes.

### Molecular Docking and Binding Pocket Analysis

The structure preparation tool in MOE was used to prepare the protein-ligand complex structures for docking via energy minimization and hydrogen bonds' adjustment (Wadood et al., 2017). The PatchDock server (http://bioinfo3d.cs.tau.ac.il/PatchDock/) (Schneidman-Duhovny et al., 2005; Mehmood et al., 2019) was labored to dock the PZase wild and mutants in a complex with PZA in order to see the impact of these mutations on the protein-drug interactions and understand the severity of such variations (Khan et al., 2018). PatchDock considers the geometric features of a ligand and receptor that are supposed to be docked. The protein complexes which maintain good interactions usually exhibit better shape complementarity. The most important region/regions in any protein structure is the active site or interacting sensitive sites on which the whole protein function depends. Variations in the amino acid sequence greatly affect the overall structural stability that often causes changes in the active site topography. Therefore, it is highly recommended to examine the impact of mutations on the active site/binding pocket of a protein as it may result in a poor or no bond formation with its ligand. Hence, we carried out a comparative inspection of the differences among the volume of a PZase wild and mutants' binding pockets. For this purpose, a web-based tool known as Computed Atlas of Surface Topography of proteins (CASTp) (Binkowski et al., 2003) was employed that can be accessed at http://sts.bioe.uic.edu/castp/index.html?3igg. It measures a curved surface area that is suppressed inside the receptors. It also calculates the total surface and volume of the sensitive site that comes in very handy while analyzing the functional consequences of mutations on a protein structure.

### Validation via Molecular Dynamics Simulation

In order to gauge the effects of PZase mutations on the protein structural and functional stability, MD simulation was carried out individually for all the wild and mutant proteins via Gromacs 5.1 (Wang et al., 2019). The simulation was performed in two phases, such as the apo and in complex with the PZA drug. The PZase protein contains Fe^2+^ as a natural ligand that cannot be removed from the protein but it is not defined in any of the Gromacs' forcefields. Thus, the topology for Fe^2+^ was generated via the PRODRG server (http://davapc1.bioch.dundee.ac.uk/cgi-bin/prodrg/submit.html). The cubic SPC simple point charge (SPC) water box was used while the boundaries were set to ≥10 Å. The system was stabilized by adding Na^+^/Cl^−^ ions. A two-step energy minimization (NVT and NPT) for a duration of 50,000 ps was continued till the steepest descent minimization was completed and the maximum force reached below 1,000 KJ/*mol*^−1^/n*nm*^−1^. An overall pressure equal to 1 bar and a temperature 300K was kept with a time gap of 2 fs to achieve a stable state. A gap of 10 steps under a position restraint condition was kept for the hefty atoms to update the list of non-bonded pairs and LINear Constraint SoLVer (LINCS) (Hess et al., 2008). The particle mesh Ewald method was used for the calculation of electrostatic interactions. In order to maintain a constant temperature inside the box, the v-rescale which is an optimized Berendsen thermostat temperature coupling technique was used. Finally, all the simulations were run via amberGS forcefield for a duration of 100 ns.

Once the MD was completed, all the obtained trajectories were examined for conformational drifts. The root mean square deviation (RMSD), root mean square fluctuation (RMSF), and the radius of gyration (Rg) were computed via the built-in Gromacs' functions such as *g_rms, g_rmsf*, and *g_gyrate*.

### Principal Component Analysis

The principal components analysis (PCA) is used for reducing the dimensions of the data obtained as a result of MD simulation. This is helpful to observe harmonic motion in a configuration space up to a minimum degree of freedom. Therefore, PCA is used to inspect the MD trajectories and in identifying the most prominent modes in the overall movement of molecules. The principal motion directions of a protein in an essential dynamics (ED) are termed as eigenvectors. The wild and mutated proteins' movement in a multi-dimensional room was observed via the vital eigenvectors that projected in the coordinates of the cartesian trajectory. A covariance matrix for all the protein structures for backbone C atoms was constructed from the trajectories that remove the translational and rotational atomic rearrangements. The covariance matrices' eigenvector and eigenvalues were calculated and the first two principal components (PC1 and PC2) were projected on the plot. The in-built Gromacs commands *g_covar* and *g_anaeig* were used to calculate PCA from the Gromacs' trajectory.

The Free energy landscape (FEL) signifies the energy states of a system. A uniform state with nominal energy can be recognized as deep valleys depicted on the graph while the intermediate conformations are the borderlines in between these deep valleys. In the present case, an in-built Gromacs option *g_sham* was used for the analysis of FEL via the following equation:

(1) $$\begin{array}{r} \Delta G\left(PC1,\;PC2\right)=-KBTlnP\left(PC1,PC2\right) \end{array}$$

Here, the reaction coordinates are denoted by PC1 and PC2, KB is the Boltzmann constant and P (PC1, PC2) stands for the initial two principal components probability dispersal of the system.

### Free Energy of Binding (FEB)

The amount of freely available energy often termed as Gibbs free energy (G) (Sugita and Kitao, 1998) for the PZase mutants was estimated and depicted against the wild MTB PZase. Constant temperature and pressure were used to minimize the ***G*** and attain a chemically stable equilibrium state. To perform a smooth process of Gibbs free energy calculation, the minimization of ***G*** is an essential step that has to be taken.

Calculating free binding energy is also necessary for understanding the association of a ligand with its' receptor. A *g_mmpbsa* (Kumari et al., 2014) module implemented in Gromacs was used to calculate various energies (Hou et al., 2010; Homeyer and Gohlke, 2012). The resulting files were processed via the available Python script (MMPBSA.PY) that computed the final energies for WT and MTs concerning the PZA drug. As a whole, simulation from the last 10 ns trajectory was used for this purpose. The following equation was solved to calculate each entity's free energy:

(2) $$\begin{array}{r} \Delta G\left(bind\right)\;=\;\Delta G\left(complex\right)-\left[\Delta G\left(receptor\right)+\Delta G\left(ligand\right)\right] \end{array}$$

Where:

Δ*G* complex = Protein-ligand complex binding free energy,

Δ*G* ligand = Free binding energy of the ligand

Δ*G* receptor = Protein-free binding energy,

Δ*G* bind = Total binding free energy,

The *G* can be expressed as:

(3) $$\begin{array}{r} G=Gbond+Gele+GvdW+Gpol+Gnpol-TS \end{array}$$

Where the standard molecular mechanics energy terms can be identified as:

*G*bond = Bonding interactions

*G*ele = electrostatic interactions

*G*vdW = vander Waals interactions

The Gpol and Gnpol are the total solvated free energies and are estimated via the generalized Born implicit solvent technique. And the TS stands for the entropic contribution which was calculated via normal mode analysis.

## Results

### MTB Culture Result

A total of 4518 TB suspect samples were obtained and processed for the presence of TB in PTRL from all the districts of the Khyber Pakhtunkhwa province. Among the TB suspect samples, 753 (16.6%) samples were detected as culture-positive (MTB). All the positive samples were subjected to PZA drug susceptibility testing for screening of PZA resistant MTB isolates.

### PZA Susceptibility Pattern

Out of 753 MTB positive samples, 69 (14.8%) were detected as PZA resistant. Among them, 52/69 (75.3%) and 6/69 (8.7%) samples were multidrug drug resistance (MDR) and extensively drug-resistant (XDR), respectively. To find the role of *pncA* mutations in PZA resistance, all the resistant MTB samples along with 26 PZA-sensitive and one MTB H37Rv as control were sequenced to screen for the mutations in the coding region (561 bp) of *pncA* (PZase).

### Mutations Screening in *pncA-*PZA Resistants

Among the 69 PZA-resistant isolates, 51 (74%) has 36 different mutations in the *pncA* gene (GeneBank Accession No. MH461111) including Asp12Ala, Pro54Leu, and His57Pro. We did not detect mutations in the sensitive isolates, except a synonymous mutation, 195C>T (Ser65Ser).

To estimate the performance of drug susceptibility testing (DST) compared with the *pncA* sequencing result, the genotypic and phenotypic data for all 69 resistance isolates were evaluated. Considering phenotype as a reference, among the 69 resistant isolates, 51 (74%) isolates showed mutations, with sensitivity of 79.31% (95% CI, 69.29% to 87.25%) and specificity of 86.67% (95% CI, 69.28% to 96.24%). To explore the in-depth molecular mechanism behind the drug resistance caused by mutations Asp12Ala, Pro54Leu, and His57Pro of PZase were investigated along with other multiple factors that might be involved in structural alterations and function.

### Binding Pocket Calculation of WT and Mutant PZase

**Figure 2:**
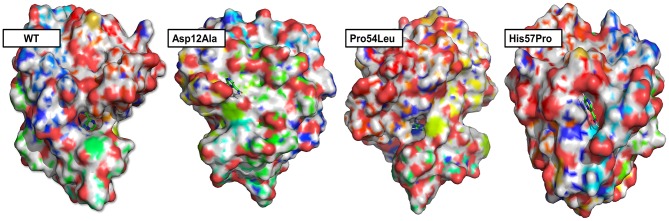
Surface analysis. The PZase WT and mutants in a complex with PZA drug are illustrated revealing alterations in the binding pocket. Significant changes in the case of Asp12Ala and His57Pro can be observed. In the case of WT and Pro54Leu, the drug remains inside the cavity while it stays on the surface in the case of Asp2Ala and His57Pro.

Any changes in the binding pockets may highly affect the linkage formation with a ligand, particularly a drug. The CASTp online tool analyzed the binding pocket to gauge the indirect effect of mutations on the structure. The pocket volume in the case of WT PZase is 585.736 Å that is considered an optimum size for the PZA drug binding. Any deviation from this volume may impede the substrate attachment. The volumes of the binding pockets for mutants PZase were compared with that of the WT, revealing consequential changes (Table 1). The surface illustrations of WT and mutants PZase have been visualized in Figure 2.

**Table 1:** Characteristics of WT and mutants PZase structures.

**PZase Mutations**	Cre-Conc ^*^ of PZA	**DST result**	**PatchDock score**	**Pocket Volume (Å)**	**ΔGbind (Total Binding Energy)**
WT	100 μg/ml	Sensitive	2386	585.736Å	−28.79
Asp12Ala	100 μg/ml	Resistant	2382	229.949Å	−23.81
Pro54Leu	100 μg/ml	Resistant	2382	229.949Å	−8.01
His57Pro	100 μg/ml	Resistant	2356	275.830Å	−19.91

* *Represents the critical concentration of PZA drug*.

### Protein-Ligand Interactions

**Figure 3:**
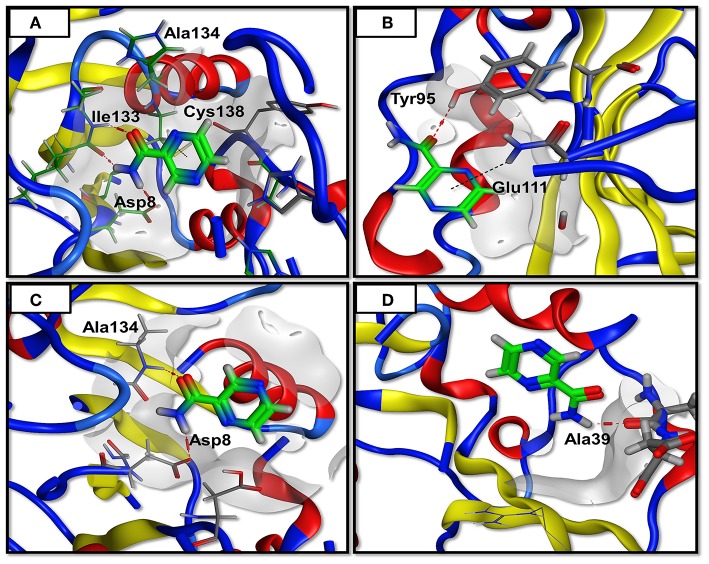
Docking analysis. The PZase WT and mutants are docked in a complex with PZA and interactions are plotted revealing loss of interactions in case of mutants. The red line denotes hydrogen bonding while the black line represents weak interactions. **(A)** WT showing three hydrogen bonding of PZA with PZase, **(B)** Asp12Ala has only one hydrogen bond, **(C)** Pro54Leu has two hydrogen bonds, **(D)** His57Pro has only one hydrogen bond.

In order to form a strong bond between receptor and a ligand, various forms of interactions such as hydrogen and hydrophobic linkages are vital. The WT formed interactions with Ala 8, Ala 134, Ile133, and Cys 138. It made 4 hydrogen bonds and one weak interaction which is far more than the mutants. The mutant Asp12Ala only formed two interactions (1 hydrogen bond and 1 weak interaction) with Tyr 95 and Glu 111. Two hydrogen bonds were established by Pro54Leu with Asp 8 and Ala 134. The mutant His57pro was observed to be highly affected by the mutation and it formed a single hydrogen bond linkage with Ala 39. These observations confirm the PatchDock analyses as WT score is observed to be higher than the mutants (Table 1). The 3D interactions of all the docked complexes are illustrated in Figure 3.

### Protein Trajectory Analysis

**Figure 4:**
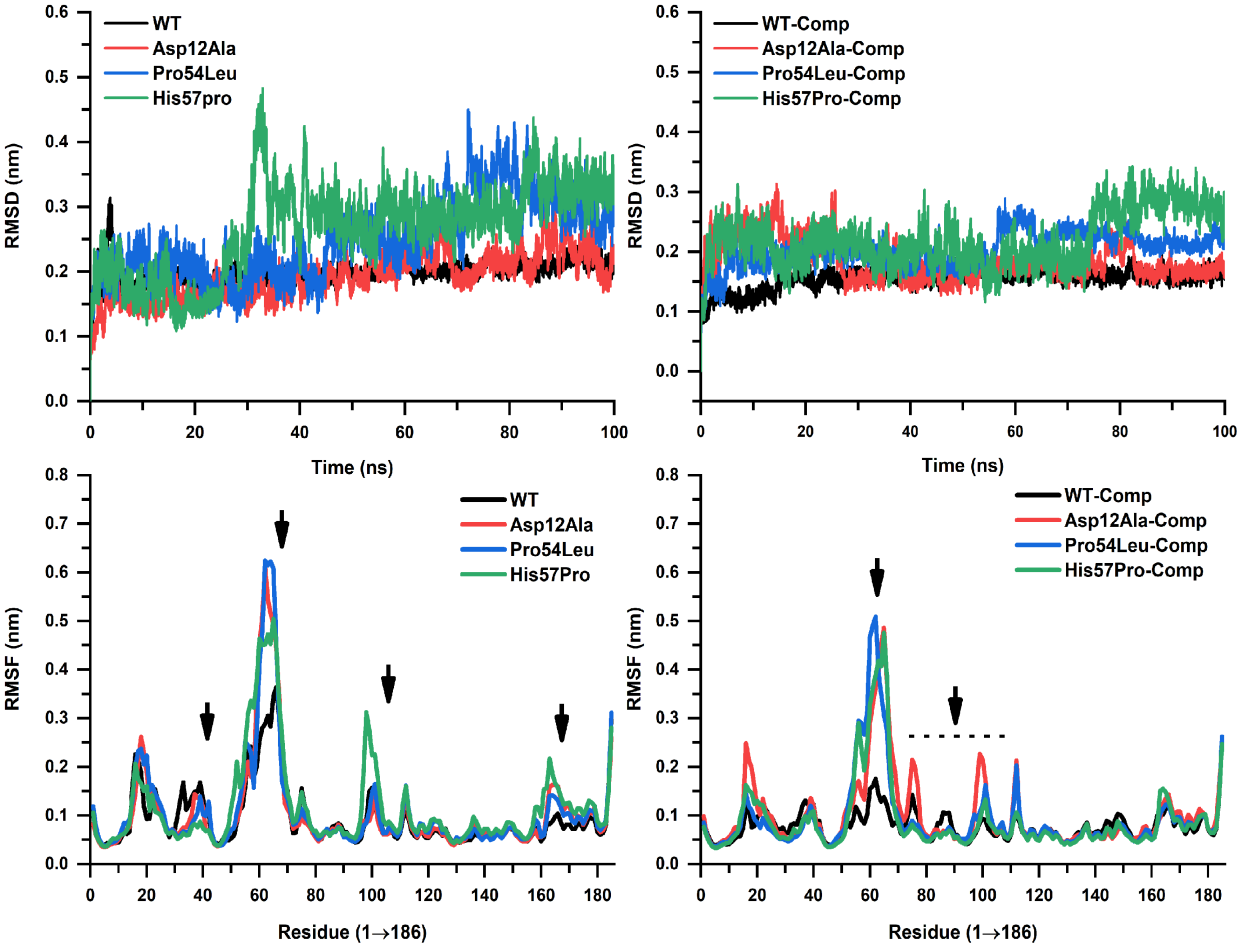
RMSD and RMSF analyses. WT and mutants in apo and complex states are showing noticeable alterations. WT is highly stable throughout the simulation while His57Pro can be seen as the most fluctuated confirmation forming only a single interaction.

MD simulation of both the WT and MTs was conducted on a high-performance cluster in apo and in a complex with the PZase drug for a duration of 100 ns in order to analyze the acquired conformational changes caused by mutation Asp12Ala, Pro54Leu, and His57Pro. The trajectories for all these three MTs along with the WT were compared and carefully examined. The PZase system for WT apo gained its stability at 30 ns while the PZase-PZA complex was observed to be stable at around 20 ns. The root-mean-square deviation (RMSD) value in case of WT apo reached up to 0.3 nm and then dropped down back to 0.2 nm where it remains fairly consistent. In the case of PZA complex, the WT shows the RMSD value of 0.14 nm with some minor fluctuations, however, it remains much stable as compared to the WT apo (Figure 4). On the other hand, all three mutations expressed notable differences in their overall stability. In the case of Asp12Ala-apo, there is not a big difference seen as compared to the rest of the MTs being reported in this study. It can be said that this mutation is the least deleterious among the three novel mutations analyzed here. However, it does not mean that this mutation does not affect protein stability. The RMSD for Asp12Ala in the first 20 ns (Figure 4) remains lower as compared to the WT. However, it goes on elevating until it matches the exact values of WT-apo PZase. The highest fluctuations in the case of RMSD for Asp12Ala can be observed in the time interval of 80–100 ns. This mutation in a complex with PZA drug (Asp12Ala-Complex) fluctuates at a much higher frequency reaching up to 0.3 nm till 25 ns from where it drops down to 0.15 nm and remains fairly stable. Though a high degree of fluctuations during 70 to 80 ns can be observed (Figure 4). The mutation Pro54Leu highly affects the stability of the protein. In its apo state, minor fluctuations are observed until 42 ns which are still higher than the WT-apo and Asp12Ala. After 42 ns, the fluctuation frequency is increased, elevating more higher than the WT and reaches 0.45 nm at about 70 ns from where it drops down a bit but keeps on changing and the scene remains the same till the end of the simulation. In the case of the Pro54Leu-complex state, substantial differences can be observed in the RMSD values.

Along with minor and major fluctuations since the start of simulation until 60 ns, it suddenly jumps up to 0.27 nm and stays there throughout the simulation. From the RMSD analysis, it was examined that the mutation His57Pro is the most deleterious mutation being reported and studied here for the first time. In its apo state, it fluctuates minorly till 20 ns where it takes a sharp leap at about 30 ns where it reaches 0.48 nm that is observed to be its highest peak. The fluctuations remain consistent throughout the simulation. Analysis of the His57Pro-PZA complex revealed that its RMSD value remains much higher than WT-complex and kept on fluctuating with minor elevations and dropdowns till 78 ns from where it takes off and reaches 0.35 nm which is its maximum point and stays there till the end of the simulation. After carefully examining RMSD values and the overall stability, it was observed that the mutation His57Pro is highly affecting the conformational stability. We believe the possible reason is that histidine at position 57 is bonded with the FE2 that is a natural ligand present in the PZase protein which aids in the overall stability of the protein. Mutating this residue into proline loses interactions between FE2 and histidine and thus the protein remains highly unstable. The mutation Pro54Leu can also be correlated with this phenomenon as it is only 2 residues away from the interaction of FE2 and lies in the same hotspot. Thus, it is also considered as a very sensitive position. The mutation Asp12Ala, on the other hand, is located far from the active site and thus could be the reason for its minimum sensitivity.

The RMSF was also calculated to observe the residual fluctuation and their dynamic behavior for the backbone. All three MTs exhibited higher RMSF values than the WT both in apo states and in a complex with PZA (Figure 4). The WT apo's RMSF value ranges from 0.4 to 0.35 nm while the WT-complex ranged from 0.05 to 0.15 nm. In the case of the mutation Asp12Ala-apo, the highest RMSF is noted at the position between residue 50 and 60 reaching up to 0.62 nm. In its complex state, the highest value observed is 0.5 nm between residues 58 to 72. The MTs Pro54Leu-apo and complex state reached 0.65 and 0.5 nm, respectively. In the case of His57Pro, the highest peak observed is 0.5 and 0.49 nm in its apo and complex state, respectively, which is lower than the rest of MTs. The other MTs remain fairly consistent with some minor and few major oscillations. The fluctuation score for a small protein is acceptable if it's below 0.2 nm (2Å). The graphical representation shows that the highest fluctuations occurred in between the residue 50 and 70, are much higher as compared to the WT. The overall stability is highly affected and the RMSF outcomes favor the RMSD conclusions.

**Figure 5:**
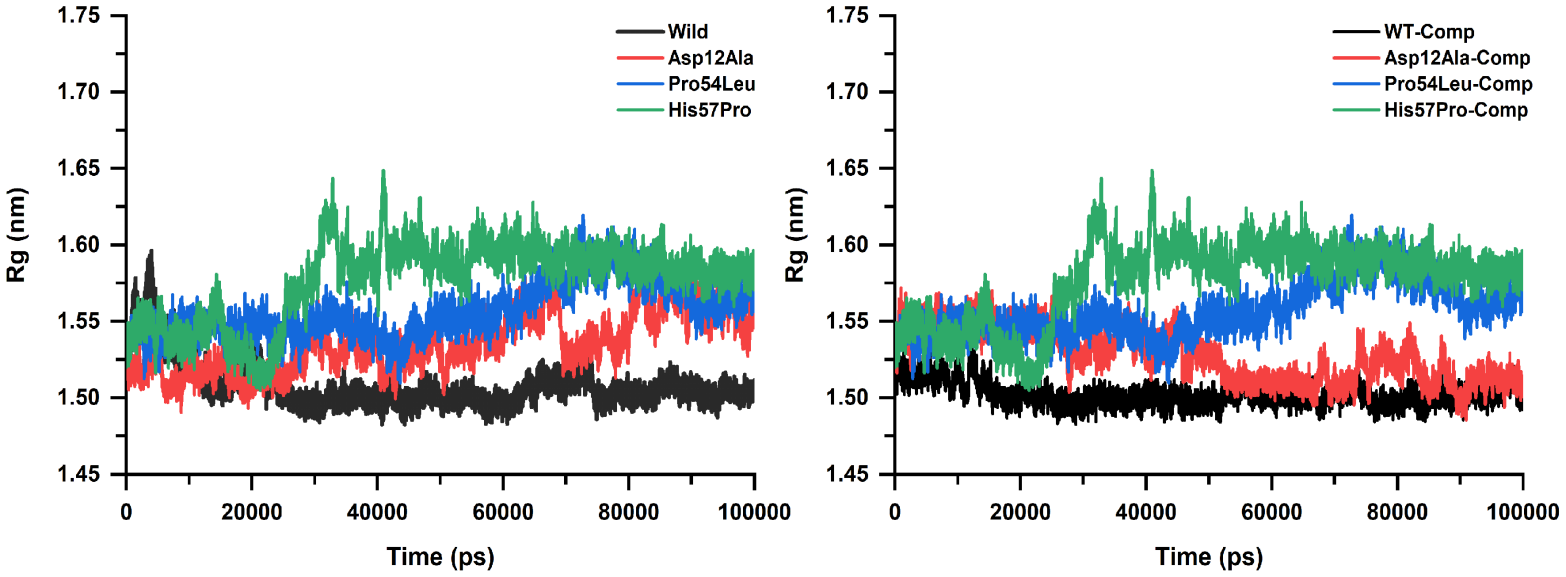
Rg analysis of WT and mutants (apo and complexes). The high amount of fluctuation is observed in the case of mutants represented in green, blue, and red color while the WT exhibits a uniform motion shown in black. A uniform Rg value express no alteration in the structural folding while the mutants remain highly unstable.

The level of structural firmness and folding was analyzed via the radius of gyration (Rg) plotted against the time (Figure 5). The insights into the overall dimensions of the protein can be provided by Rg and thus it is a necessary constraint for defining the conformational solidity of the total protein system. The Rg results were similar to that of the RMSD and RMSF analysis in the sense that these mutations expressed substantial deviation from the WT protein. The Rg curves for mutation Pro54Leu and His57Pro were observed to be much higher both in the apo and their complex states. It is important to mention here that no major change was observed in the individual values both in their apo and complex states. However, an important difference in the apo and PZA-bound complex in the case of Asp12Ala was observed. In its apo situation, it kept on elevating with major fluctuations while in its complex state, it remains fairly stable.

### Essential Dynamics Simulation Analysis

**Figure 6:**
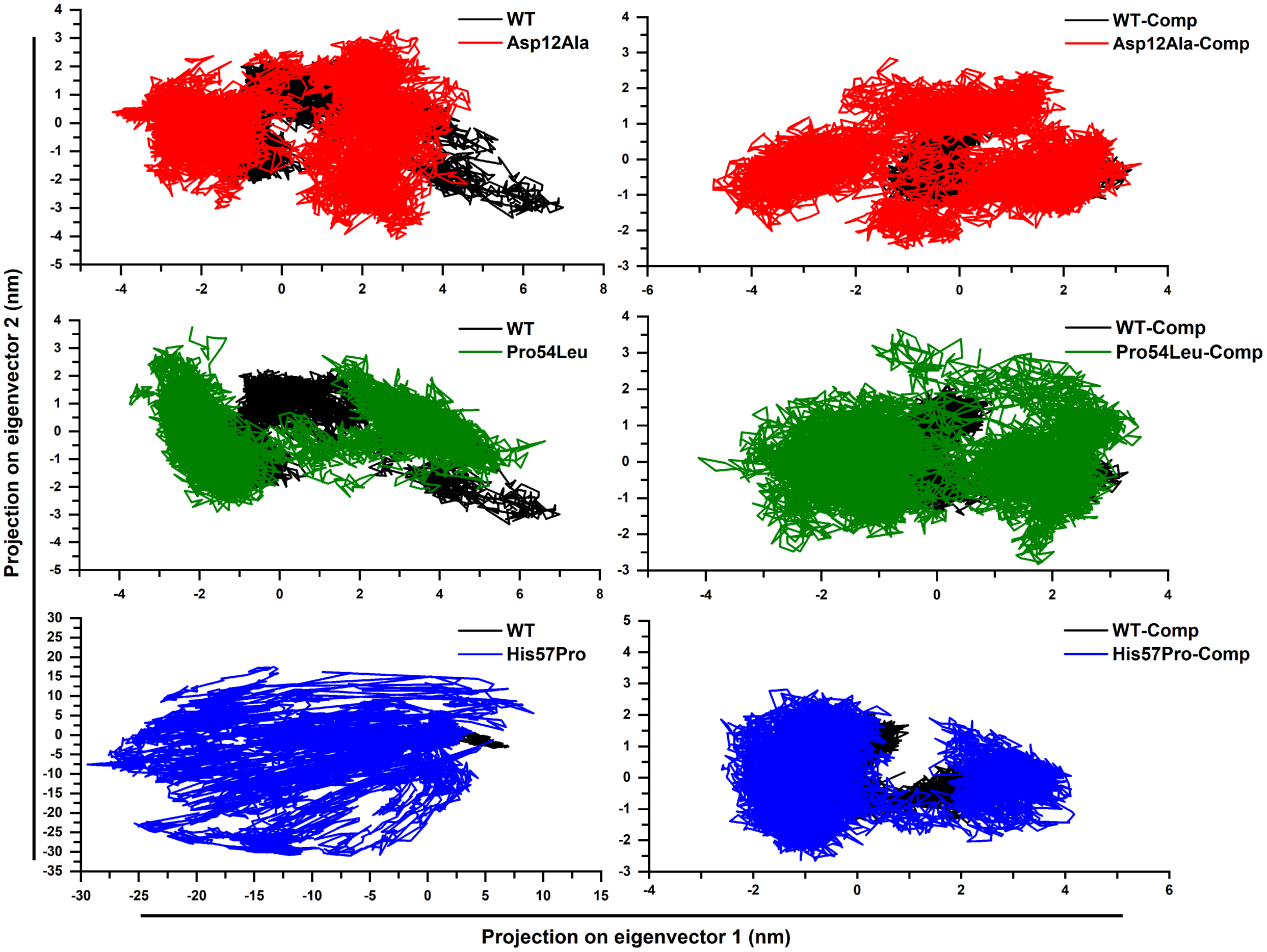
PZase WT and mutants' PCA. Deviation from the native motion can be observed in the case of mutants and that are more scattered as compared to the WT. The black color represents WT while the mutants are shown in blue, green, and red.

Upon the analysis of PCA (Figure 6), a big difference among the WT and MTs was observed. The mutant structures both in the case of apo and complex states covered more areas than the WT. It was also observed that the motion of mutant structures was quite uneven and random as compared to the native PZase structure which signifies the difference in the dynamics of atoms (uncorrelated). In the case of WT apo, the PCs lies in between −3 to 2.5 on PC2 and −2 to 7 on PC1 while in its complex state, the motion ranges from −1 to 2.0 on PC2 and −1.5 to almost 3 on PC1. Apart from this, the PCs were observed to be more compact and evenly traversed along the scale.

In the case of apo Asp12Ala and Pro54Leu, a noteworthy deviation from the WT was observed. The apo Asp12Ala ranged from −4 to 3.5 on PC2 and −4.3 to almost 5 on PC1. The apo Pro54Leu ranged from −3 to 3.9 on PC2 and −3.9 to almost 7 on PC1. The highest difference was observed in the case of apo His57Pro that was observed to be extremely uncorrelated and ranged from −30 to 17.5 on PC2 and −29.5 to 10 on PC1. The reason for this motion as mentioned earlier is the fluctuation caused in the active site of the protein that results in loss of ligand binding capability and the overall system remains highly unstable. All the mutations in complex states also deviated and scattered in various directions. The PCs in complex states were relatively less scattered when compared to the apo states.

**Figure 7:**
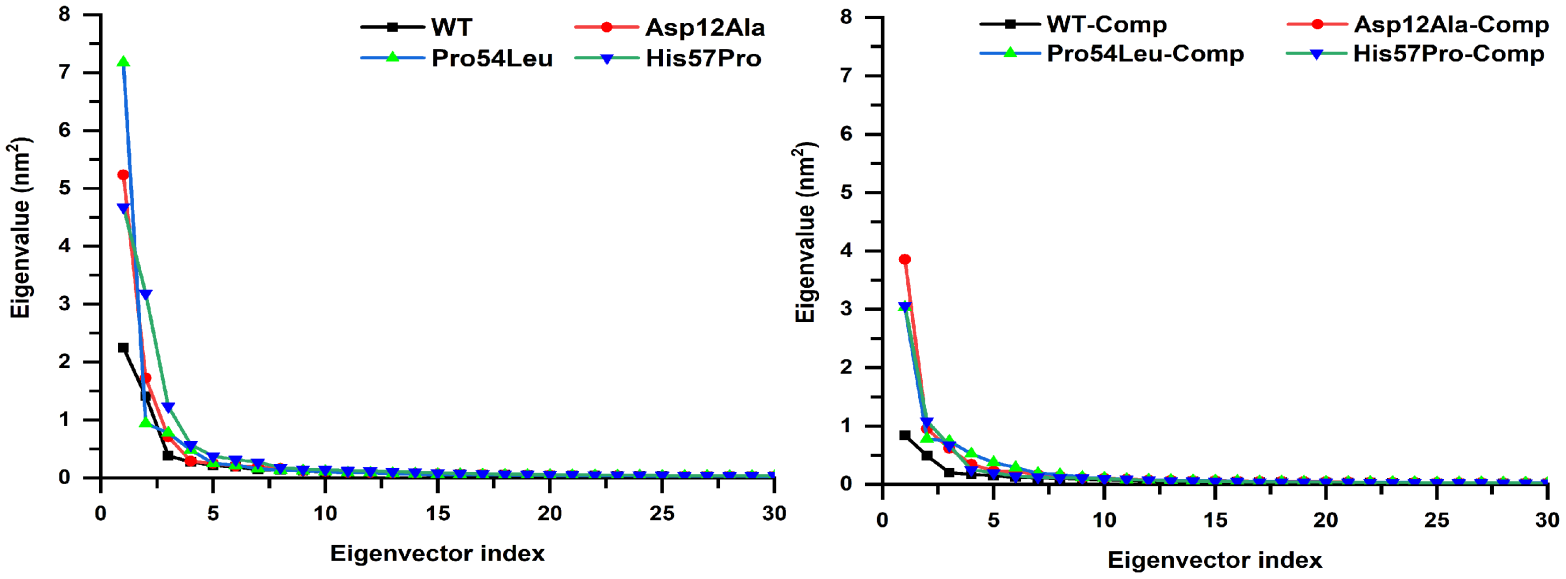
Projecting the dynamics of protein along eigenvectors. The divisions of the initial 30 eigenvectors drawn against the corresponding marks obtained from the total trajectory of the MD simulation. The WT scales much lesser than their mutants.

A multi-dimensional least square fit is involved in the projection of motion of a trajectory. The correlation for a combined configuration in a particular direction is represented by the first eigenvector with the best fitting value. Garcia applied these multidimensional fitting in the dynamics of proteins. This represents the direction that fits best in accordance with the number of eigenvectors (García, 1992). A high amount of variation was observed demonstrated by the initial four eigenvectors that tailed a fixed value since the tenth eigenvector. All these PCA analyses revealed the uncorrelated motion of MTs and proved that PZase MTs occupy more space as compared to the WT (Figure 7).

### FEB Calculation

**Figure 8:**
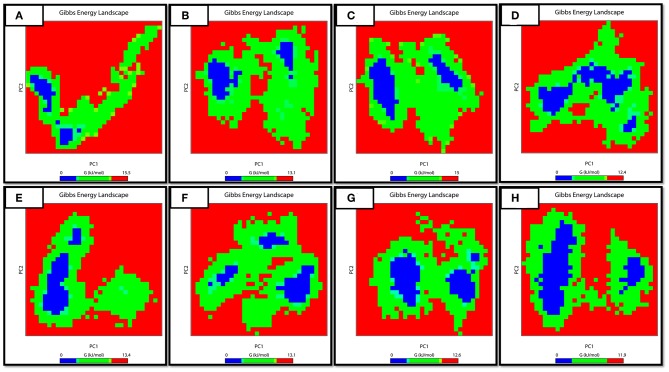
FEB Landscape. The free energy of binding landscape for wild and mutated proteins in their apo and wild states is depicted along with their value bars against PC1 and PC2. A noticeable difference can be observed. The red color represents the highest energy state. (**A**: WT-apo, **B**: Asp12Ala-apo, **C**: Pro54Leu-apo, **D**: His57Pro-apo, **E**: WT-Comp, **F**: Asp12Ala- Comp, **G**: Pro54Leu- Comp, **H**: His57Pro- Comp).

The work done during the exchange of heat by a closed system with its surroundings is measured via FEB. It is also an important approach for studying the protein structure-function relationship. Changes in values for FEB might have importance in calculating the stability of the considered proteins' confirmation. In order to explore the protein conformational shift from WT to mutant, the FEB for the first two principal components (PC1 and PC2) was calculated. The free energy of binding landscape for both the apo and complex states of WT and three MTs are shown in Figure 8.

In the case of apo simulation, it was observed that the energy values ranged from 0 to 15.05, 0 to 13.01, 0 to 15.00 and 0 to 12.01 kJ/mol for WT and three MTs (Asp12Ala, Pro54Leu, His57Pro) protein, respectively. Similarly, for the bound states of PZase, the values ranged from 0 to 13.04, 0 to 13.01, 0 to 12.06 and 0 to 11.09 for WT, Asp12Ala, Pro54Leu, and His57pro, respectively. The minimum energy area is indicated by a blue color. From the figure, WT protein showed a clear large single global energy minima basin (in blue), whereas the three mutant proteins showed several different energy minima states. The smaller blue areas suggest more stability of protein while more blue areas indicate transitions in the protein conformation followed by the thermodynamically more favorable state. The WT is having very low energy as compared to the reported mutations. It means that the native structure has a more stable cluster as compared to the mutant structures that might be involved in low binding affinity with PZA, causing resistance.

**Figure 9:**
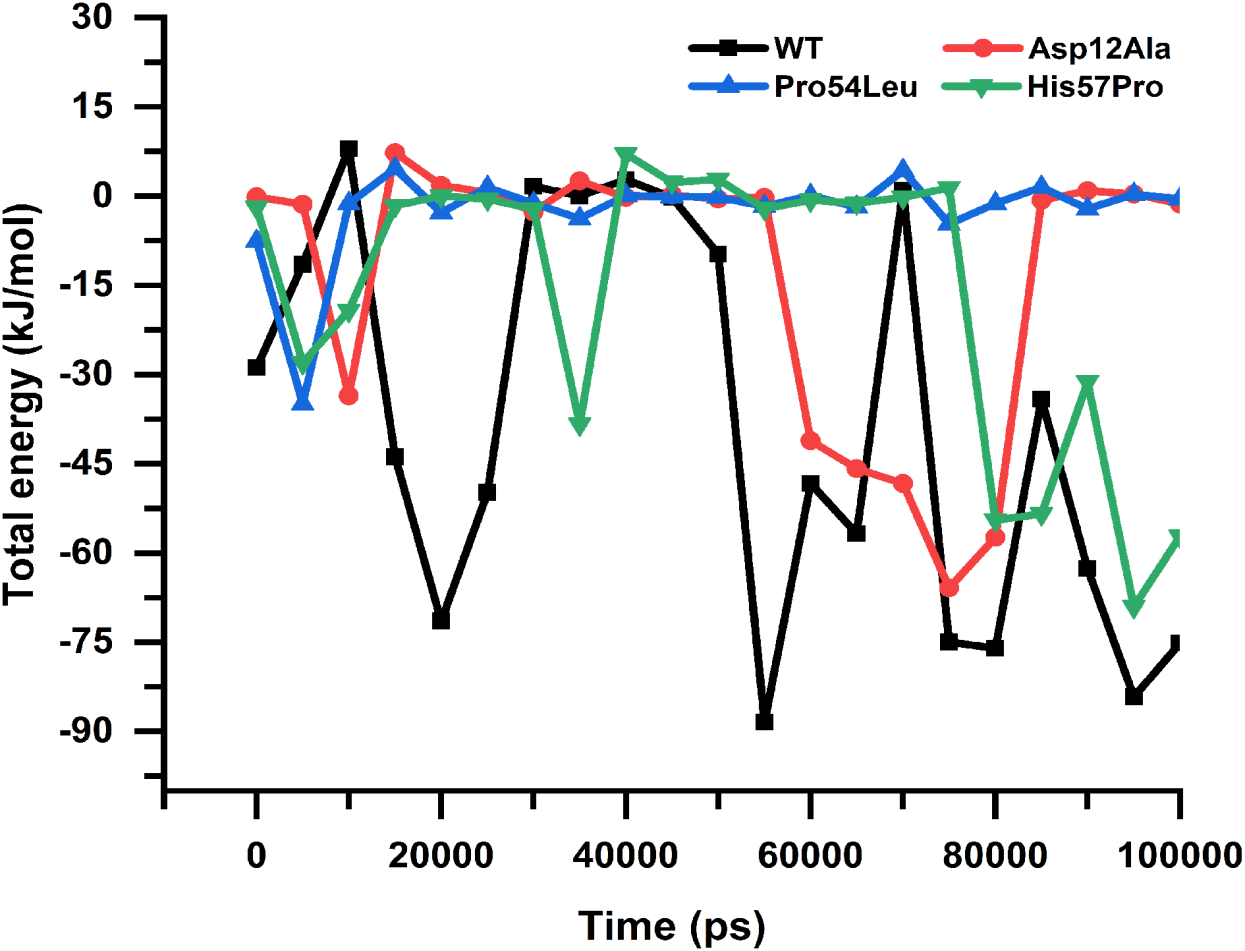
Protein-drug total energy. WT and mutants can be observed showing a noticeable difference in the total drug-protein energy till the end of the simulation. The mutant Pro54Leu is observed to be having uniform energy; however, in the case of Asp12Ala and His57Pro, the energies are more unstable.

The free energy calculation for all the MTs and WT revealed major differences in their binding capabilities with their ligand (PZA). The *g_mmpbsa* function of the Gromacs exported three different files that contain information about apolar solvation energies such as vdW, Electrostatic and protein-drug total energy. To obtain the final change in the energy, the output data files from *g_mmpbsa* were handled by a given python script which exported the final values (Table 1). The total energy for the WT and MTs was also plotted against time to present a visual view of the energy' change along the time (Figure 9).

### Distance Matrix

**Figure 10:**
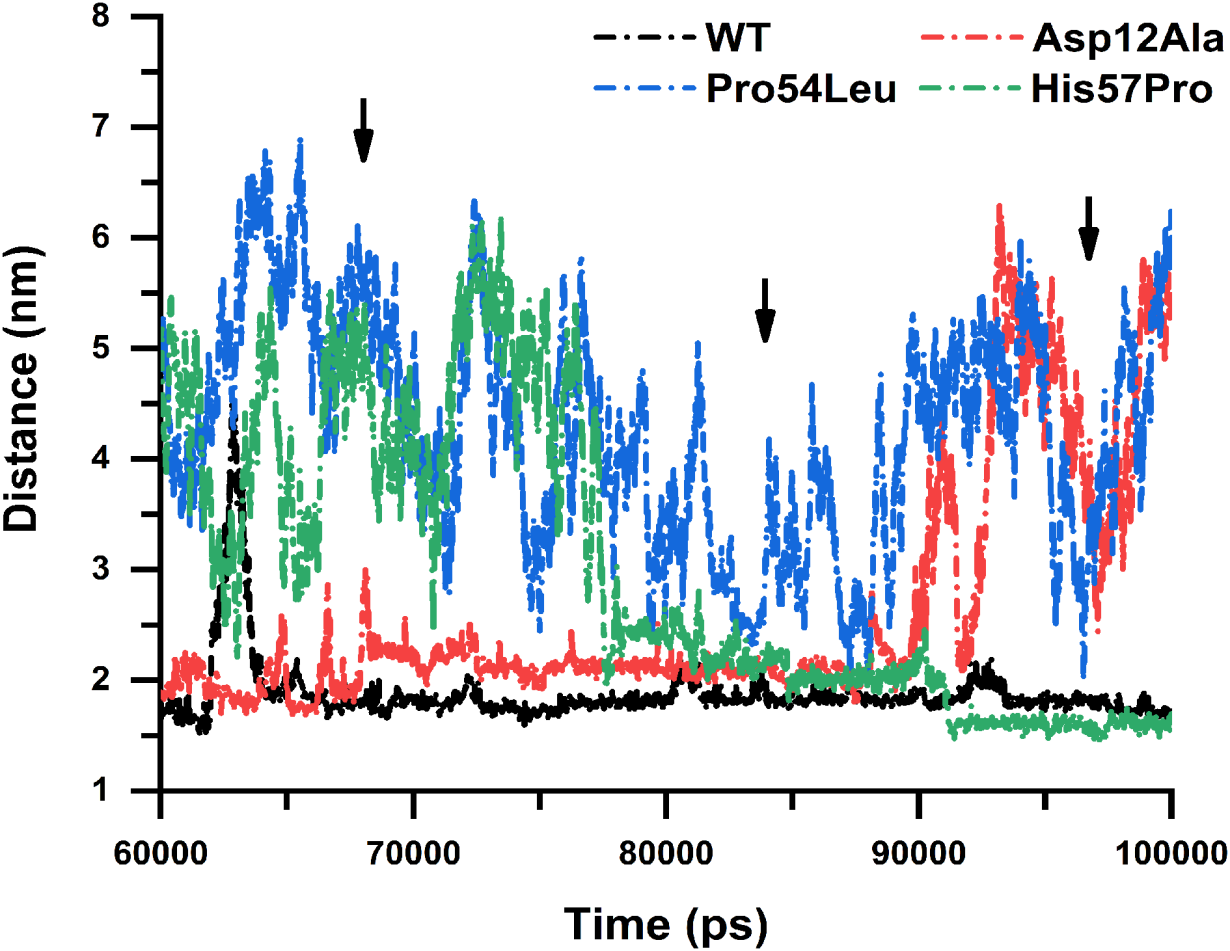
Distance stability analysis. WT and mutants showing the distance stability calculated between the receptor and PZA, revealing elevated instability in the case of Pro54Leu and His57Pro. The Asp12Ala represented in red is comparatively quite stable while the WT is expressing a uniform nature till the end of the simulation.

The distance stability between two interacting residues bears great importance because it could directly affect the interactions to be established or lost. The average distance between a WT PZase and drug is almost uniform except for some major fluctuations that occurred in the middle and at the end of the simulation. We calculated the distance between the drug and all three MTs including the WT for the last 60 ns of simulations to inspect how the stability of the distance is affected. There was a great difference between the WT and MTs regarding their distance stability and it was observed that MTs highly fluctuated till the end of simulation while the WT was highly stable, yielding a straight line (Figure 10).

Similar to the previous analysis carried out in this work, the mutation Asp12Ala is comparatively much more stable but the MTs Pro54Leu and His57Pro drastically affected the distance stability between PZase and PZA. This signifies the impact of these mutations on the binding affinity of PZA and proves how they cause the PZA drug resistance.

### Covariance Analysis

**Figure 11:**
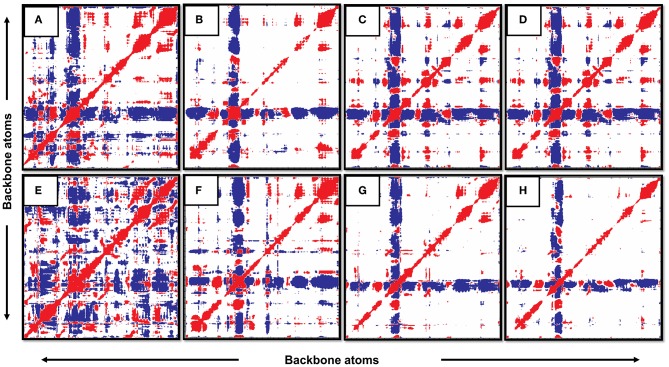
Covariance analysis. The WT in its apo and complex states show more correlated motion than its mutants. (**A**: WT-apo, **B**: Asp12Ala-apo, **C**: Pro54Leu-apo, **D**: His57Pro-apo, **E**: WT-Comp, **F**: Asp12Ala- Comp, **G**: Pro54Leu- Comp, **H**: His57Pro- Comp).

Analyzing ED can help to measure the receptor's large-scale motion. For all the apo and bound states of PZase, the covariance was calculated via the in-built *g_covar* function that rendered the *.xpm* file which was converted to .eps format and adjusted via the Photoshop application. The red and blue colored regions represent the correlated (positive) and anticorrelated (negative) motion of atoms. Visual inspection of the following figures reveals that the WT PZase atoms mainly exhibit correlated motions. A large-scale motion of the receptor can be measured through the analysis of ED. Positive (correlate motions) and negative motion (anticorrelated motions) of the backbone atoms are shown by red and blue regions, respectively. The red and blue are the positive and negative regions. WT PZase atoms were mostly involved in correlated motions [Figure 11A (apo) and Figure 11E (complex)] in comparison with MTs [Figures 11B–D (apo) and Figures 11D–H (complex)], which revealed more anti-correlated gesticulations.

It can also be observed that the motion of atoms in the native states is more compact while the MTs expressed a very distorted form of the backbone atomic motion. The conclusion from this investigation also confirms the previous deductions based on various analyses carried out in this study. Hence, it can be claimed that these reported mutations are exceedingly deleterious and highly responsible for the PZA drug resistance.

## Discussion

The rise of first- and second-line drug resistance is a foremost barrier toward the WHO plan of TB free world by 2035 (Gilpin et al., 2018). The first line of defense is the PZA drug that is highly useful in the execution of non-replicated MTB's subpopulations. Previous reports on the PZA resistance claimed that *pncA* gene mutations are responsible for the development of drug resistance. Nevertheless, in infrequent situations, it is observed that *rpsA* and aspartate decarboxylase (panD) genes mutation may also cause resistance to this first line of defense (Shi et al., 2014; Akhmetova et al., 2015). Recently we identified some novel mutations in the *pncA* gene that are PZA resistant isolated from the Northern areas (KP) of Pakistan. The data is uploaded to NCBI and can be accessed via the accession number MH461111-17 in the GeneBank. Although these novel mutations are identified and passed through several wet lab investigations that confirmed the resistance and importance of these mutations, the molecular mechanism behind these mutations (Asp12Ala, Pro54Leu, and His57Pro) was still veiled. Therefore, the motivation for the current study came from these resistant mutations with unknown mechanisms. Hence, the present work was designed to address several questions regarding these mutations such as the level of structural changes, alterations in the binding patterns and energies, and how they cause resistance. A high-performance computational study was conducted to explore the mechanism underlying the effects caused by these variations.

The sequence of amino acids defines the 3D conformation of a protein which is crucial for the native function of the protein and latter for the health of the cell. However, changes in this amino acid sequence may cause minor or major conformational drifts that could directly affect the protein's native function because the function of the protein is highly dependent upon the structure.

These variations could greatly affect the flexibility of a protein and such deviation from the native position could make it really hard for the desired ligand to be bound. Because this has a direct effect on the interactions that have to be established between these two entities. Here we used MD simulation that plays an important role in carrying out such analysis and its applications can be further expanded by improving its force fields that will result in more accurate analysis. Studying a protein-ligand interaction via MD is a widely applied technique that is used for various purposes such as computational drug designing or analyzing proteins' dynamics. One of the reasons for its popularity and usability is its accessibility and the results are comparable with the *in vitro* investigations. For instance, in case of studying the drug resistance, a crystal structure is analyzed. However, the crystal structure for every protein-ligand complex is not available and even if it is available, the mechanism cannot be explored. Therefore, MD simulations have a great advantage over experimental procedures in exploring the detailed mechanism regarding a particular situation such as drug resistance in this case. One of the main use of MD analysis is the confirmation of docking results. Molecular docking is a common technique that is used to observe the interactions between a ligand or drug with a receptor (protein). But, the docking results alone are not sufficient for making a scientific decision because of various missing factors such as aqueous medium, standard energy minimization, temperature, and pressure. Therefore, the obtained outcomes as a result of molecular docking are further validated by MD simulation which is more advanced and reliable. Based on the MD analyses, for all mutations, the RMSD and RMSF values of MTs (Asp12Ala, Pro54Leu, and His57Pro) appeared to be more elevated in contrast to the WT which indicates a high level of instability. These outcomes agree with the previous reports [1–4] on PZase mutations (Figure 4) (Vats et al., 2015; Aggarwal et al., 2018; He et al., 2018). It is suggested that variations in the sensitive site residues may not only modulate the allosteric site but also cause drug resistance.

For a protein, its compactness and folding are highly important which is estimated via Rg that is the mass-weight root mean square distance of atomic assembly from a mutual point of mass, plotted against time (Figure 5) (Lobanov et al., 2008; Smilgies and Folta-Stogniew, 2015). The Rg values altered highly in the case of MTs throughout the simulation period which indicates an unstable pattern of protein while the WT yielded a very uniform Rg graph that represents a stable protein folding. This protein folding instability caused by mutations in the case of PZA resistance is already confirmed by Vats et al. (2015) who explored PZA resistance in case of mutation K96R that caused enlargement of the binding cavity.

Apart from structural compactness and RMSD analysis, the docking approach was also used to have a clear picture of the changes in the binding mode of PZA. A noteworthy difference can be found in the score, electrostatic interactions and most importantly, the hydrogen bonding (Figure 3). WT PZase established more hydrogen bonds than the MTs. Biding pocket volume may directly affect the binding capability which is also estimated and changes are observed especially in the case of His57Pro. Histidine at position 57 is an important interacting residue in the PZase pocket that is replaced by proline resulting in a highly unstable configuration and loss of interactions with the PZA drug.

The drug has to be tested and scored on various mutations especially those which occur in the vicinity of the binding pocket as they cause more turbulence in the overall structural configuration. This will be greatly helpful in overcoming the emerging situation of MTB cases. The metal ion displacement and effects on the hydrogen bonding also have to be considered in further studies. The obtained outcomes in a combination with similar studies regarding mutations and MTB drug resistance along with other similar proteins can also be used for training a computational model that could help in analyzing the deleterious nature of a particular mutation and its consequences. Furthermore, an *in vivo* model can be used to incorporate these mutations and analyze their role in drug resistance individually. Similarly, new drugs predicted by machine learning approaches or other experimental techniques can be tested on the mutated model to gauge its engagement with the target and observe the survival. Variations that reduce the interactions formed between the metal ion and protein may be treated by using a nanoparticle in addition to the drug that could enhance the drug efficiency and stability of the protein. For this purpose, ions that share the same chemical properties with the Fe^2+^ would be more suitable.After carefully examining the effect of mutations, Asp12Ala, Pro54Leu, and His57Pro, we have concluded that these mutations might be involved in affecting PZase activity, flexibility, stability, and causing high fluctuation in the native motion of the structure. As a whole, the performed analyses greatly support the hypothesis regarding these mutations being responsible for drug (PZA) resistance. Thus, this study may help understand the overall PZase activity and in proposing new drugs to improve the management of TB drug-resistance and cope with the current situation.

### Tools and Resources Used

**Table:** 

**Tool**	**Algorithm/Function/coding environment**	**Purpose**	**Accessibility**	**References**
MOE	Protein builder, Amber12EHT, Protonate3D, MOE_Dock, London dG	Drug discovery & visualization	https://www.chemcomp.com/Products.htm	Wadood et al., 2017
CASTp	Delaunay triangulation & discrete flow	3D surface prediction	http://sts.bioe.uic.edu/castp/index.html?2cpk	Binkowski et al., 2003
PyMOL	OpenGL Extension Wrangler Library (GLEW) & Poisson–Boltzmann equations	Molecular visualization	https://pymol.org/2/	Bhattacharya et al., 2018
Gromacs	leap-frog algorithm	Molecular dynamics package	http://www.gromacs.org/	Wang et al., 2019
PubChem	HTML	Chemical molecules' database	https://pubchem.ncbi.nlm.nih.gov/	Kim et al., 2015
PatchDock	Geometry-based molecular docking	Molecular docking server	http://bioinfo3d.cs.tau.ac.il/PatchDock/	Schneidman-Duhovny et al., 2005
RAMPAGE	PHP	Ramachandran plot analysis	http://mordred.bioc.cam.ac.uk/~rapper/rampage.php	Lovell et al., 2003
RCSB PDB	HTML	Database of 3D macromolecules	https://www.rcsb.org/	Berman et al., 2000
UCSF Chimera	Amber ff14SB, Python	Interactive visualization & analysis	http://www.cgl.ucsf.edu/chimera/	Banu et al., 2018
Adobe Photoshop	Nearest Neighbor, Bilinear, Bicubic, Bicubic smoother, Bicubic Sharper	Raster graphics editor	https://www.photoshop.com/	www.adobe.com

## Data Availability Statement

All data generated and analyzed during this study are included in the article. The raw datasets are available from the corresponding author upon a reasonable request.

## Ethics Statement

The studies involving human participants were reviewed and approved by Institutional Ethics Committee of Cust Islamabad and Provincial Tuberculosis Reference Laboratory (PTRL) Pakistan. The patients/participants provided their written informed consent to participate in this study.

## Author Contributions

AM and D-QW conceptualized the study. AM curated the data, experimental work was carried out by ASK and MK. AM and MK did all the formal analysis. D-QW, AM, and ACK performed the necessary investigations. The methodology is designed by AM, MK, and ACK while the funding acquisition, resources, supervision, and project administration goes to D-QW. The final validation is done by AM, ACK, and MK. The original draft is written by AM and ACK while ASK and MI assisted in reviewing the final draft.

### Conflict of Interest

The authors declare that the research was conducted in the absence of any commercial or financial relationships that could be construed as a potential conflict of interest.
